# Gastroprotective Effect of Quercetin and Misoprostol in Ethanol-Induced Gastric Ulcer in Rats

**DOI:** 10.5152/tjg.2024.24209

**Published:** 2024-11-01

**Authors:** Zheen Aorahman Ahmed

**Affiliations:** Department of Pharmacology and Toxicology, University of Sulaimani College of Pharmacy, Sulaimani, Iraq

**Keywords:** Gastric ulcers, quercetin, misoprostol, ulcer index, antioxidant effects, anti-inflammatory effects

## Abstract

**Background/Aims:**

This study aimed to evaluate the possible synergistic gastroprotective activity of quercetin and misoprostol in gastric ulcers induced by ethanol in rats.

**Materials and Methods:**

Male Wister albino rats were allocated into 6 groups: Negative control, positive control, esomeprazole, quercetin, misoprostol, and a combination of quercetin and misoprostol. All the treatment groups except for the negative control were challenged with a single dose of ethanol (90%) after 14 days of treatment. The animals were euthanized 1 hour after ethanol administration, and the blood samples were collected and used for the measurement of catalase, GSH, LDH, and IL-6. The stomachs were immediately removed and used for the measurement of gastric ulcer index, lesion area, gastric volume, and pH. Finally, gastric tissue was sent for histopathological examination.

**Results:**

The combination of quercetin with misoprostol resulted in a comparable effect to esomeprazole regarding the inhibitory effect on gastric lesions, ulcer index, and free and total acidity. Moreover, this combination significantly decreased the level of LDH with a non-significant decrease in the IL-6 level. Esomeprazole and the combination group restored the level of catalase and quercetin alone and in the combination group elevated the level of GSH. Additionally, remarkable protection appeared in the pathological findings, especially in the group treated with quercetin and misoprostol.

**Conclusion:**

This study clearly revealed the gastroprotective effect produced by combining quercetin with misoprostol by decreasing ulcer area and index, restoring antioxidant enzyme levels, and ameliorating inflammation. These findings suggest the use of this combination in a clinical setting.

**Main Points**

- Every year, 4 million people worldwide suffer from peptic ulcer disease (PUD), which has a lifetime prevalence of 5-10% in the general population.
- It has been shown that quercetin has a protective effect against gastric mucosal lesions induced by ethanol through attenuation of lipid peroxidation and an increase in the activity of antioxidant enzymes.

- Combination of quercetin and misoprostol shows gastroprotective activity by decreasing ulcer area and index, restoring antioxidant enzymes, and ameliorating inflammation.

## Introduction

One of the most prevalent digestive disorders in the 21st century is thought to be gastric ulcers. Peptic ulcers are long-lasting, frequently solitary sores that can develop anywhere throughout the digestive tract.^1,2^ The etiology of this disease is multifactorial; bacterial infection and loss of prostaglandin induced by the chronic use of nonsteroidal anti-inflammatory drugs (NSAIDs) are among the most important contributing factors. Others, like cigarette smoking, chemotherapy, radiation, hypergastrinemia, viral infection, gastric obstruction, and malignancy, also cause gastric ulcers. In all these etiologies, loss of the protective mucosal layer is the common cause of gastric ulcer.^3^ Additionally, the imbalance between the protective factors, such as sufficient blood flow and prostaglandins, and the destructive factors, like acid and pepsin, may also contribute to the etiology of the disease.^4^ Furthermore, alcohol ingestion and stress may increase the prevalence of the disease.^5^

Many medications, such as proton pump inhibitors, H2 blockers, prostaglandin analogs, and antibiotics, successfully controlled gastric ulcers. However, searching for newer, more efficacious agents with fewer adverse effects is still an interesting area for researchers.^6^ Nutraceuticals are an important source for the prevention and treatment of gastric ulcers. Various mechanisms contribute to ethanol-induced gastric ulcers. The presence of ethanol in the gastrointestinal tract produces damage to the gastric mucosa by attracting inflammatory cytokines, which in turn facilitate the generation of reactive species, producing oxidative stress and aggravating gastric tissue damage.^7^ Quercetin is a flavonoid present in various plant species and is broadly found in different vegetables and fruits. It has been shown that quercetin possesses a protective effect against ethanol-induced gastric mucosal lesions by attenuating lipid peroxidation and increasing the activity of antioxidant enzymes.^8^ The prostaglandin analog misoprostol protects against gastric lesions by inhibiting acute gastric mucosal damage induced by irritants such as boiling water and alcohol. By exerting a direct influence on parietal cells, it prevents the release of gastric acid both primarily and in response to food, histamine, gastrin, and coffee.^9^ The combination of this well-proven gastroprotective agent with natural products could be a promising line for research. Therefore, this study aimed to estimate the possible protective effects of quercetin, misoprostol, and their combination in gastric ulcers provoked by ethanol.

## Materials and Methods

### Experimental Animals

The rats used in the current study were Wister albino males weighing (160-200 g), and they were obtained from the College of Pharmacy, University of Sulaimani. The rats were housed in standard conditions in the animal house in plastic cages with regular lighting, temperature controls (25 ± 2°C), and humidity (55 ± 5%), with a twelve-hour light-to-dark cycle. The animals had unlimited access to water and were fed with a conventional pellet diet. Prior to starting the experiment, the rats were reserved for 7 days for acclimatization. The experimental protocols received approval from the Ethical Committee of the College of Pharmacy, University of Sulaimani, with certificate number (PH33-21) on November 14, 2021.

### Study Design

Thirty rats were used in the current study and were randomly allocated into 6 groups, each of 5 rats:

1. Negative control group: This group received distilled water (D.W.) via the oral route using a gavage tube daily for fourteen days.
2. Positive control group (ethanol-treated group): this group was treated with D.W. by the oral route daily for fourteen days; on day 14, it received 1 mL of 90% ethanol (Merk, absolute, suitable for HPLC, ≥99.8%) for the purpose of inducing a gastric ulcer.
3. Esomeprazole group: received 30 mg/kg esomeprazole (supplied by Pioneer Company for pharmaceutical industries- Iraq) by oral route daily for 14 days with ethanol administration.
4. Quercetin group: received 50 mg/kg quercetin (powder from Sigma, HPLC grade) by oral route daily for 14 days with ethanol administration.
5. Misoprostol group: received 100 mg/kg misoprostol (Pfizer Cytotec) by oral route daily for 14 days with ethanol administration.
6. Misoprostol + quercetin group: received misoprostol 100 mg/kg + quercetin 50 mg/kg orally daily for 14 days with ethanol administration.

On the last day, the animals were euthanized 1 hour after ethanol administration, and the blood samples were collected by cardiac puncture and sent for measurement of biochemical tests.

### Gastric Lesions Assessment

After sacrifice, rapid removal of the stomachs was done, opened along, and washed using normal saline solution (0.9% NaCl). Then the stomachs were fixed on a tile. Lesion measurements were performed using a Java-based image processing program (ImageJ); the following equations^10,11^ were used for calculating the Ulcer index and the percentage Ulcer Inhibition of (UI):

UI = [ulcerated area (mm^2^) / total area of the gut (mm^2^)]

The level of ulcer inhibition was calculated and expressed as a percentage as follows:

% UI = (Ulcer index _Positive_ _Control_ –u -lcer index _Test_) × 100 / ulcer index _Positive_ _Control_

### Estimation of Gastric Volume and pH

A volumetric cylinder was used to collect the gastric content.^12^ After centrifugation (4000 rpm/10 min), the supernatant was taken and used for the measurement of the volume and the pH by a digital pH meter.^13^

### Measurement of Total Acidity

Total and free acidity was determined by titrating the gastric juice with 0.1 N NaOH, and methyl orange reagent was used as an indicator by producing a salmon color secondary to neutralization of the free hydrochloric acid. Phenolphthalein served as the indicator for measuring total acidity. Then, to the gastric juice (1-2 drops) of methyl orange reagent was added. The indicator of the free acidity was the appearance of a bright red color. The titration continued with 0.1 N NaOH till a canary yellow color appeared. Free HCL was represented by the volume of NaOH. Then, the titration continued with NaOH after adding 1-2 drops of phenolphthalein until the red color appeared. The number of mL of NaOH used for the titration represented the total acidity.^12^

*Y* = 0.1 N NaOH (mL) × 10

where *Y* = total acidity (m Eq/L)

### Biochemical Tests

On day 14, blood samples were collected from the animals by cardiac puncture. After centrifugation (3000 rpm for 10 minutes), the serum was used for measuring LDH, Catalase, GSH, and IL-6 using an ELISA kit (Bioassay Technology Laboratory, UK).

### Histotechnique Procedure

At first, before starting with the sacrification protocol, animals were fasted for 1 day. After sacrification, animals were dissected, and tissue samples were collected for histopathological procedures. Immediately after the necropsy, gastric tissue samples were cleaned and washed with normal saline solution, then secured with 10% formalin for 2-3 days. Subsequently, the sections were positioned and rendered immobile within plastic cassettes specific for tissues that underwent dehydration by an increasing ethanol alcohol series, followed by 3 stages of xylene clearance. Subsequently, the stomach tissues were infiltrated and fixed using a wax embedder at a melting temperature of between 60°C and 70°C in molten paraffin blocks. A rotary microtome was used to segment embedded tissues at a thickness of 5 µm. Tissue slices were then dried and adhered to glass slides. Subsequently, the tissue slices underwent 30 minutes of xylene solution cleaning and deparaffinization, followed by 5 minutes of hot plate drying. After applying Harris’s hematoxylin and eosin solution to stain the tissue sections, they were washed with xylene, covered, and seen under a microscope.

### Scoring of the Lesion

Generally speaking, in 10 randomly chosen fields, mucosal erosion was measured and estimated as a percentage of computed erosional length and depth in µm, while the exudate and the edematous area were measured and expressed as a mean percentage. Furthermore, using high-power magnification (100X), 5 distinct fields were used for counting the inflammatory cells and necrotic debris. The mean average was then statistically assessed as a percentage. Ultimately, the lesion scoring-grading system was developed based on the mean of all morphometric values: 0-10% represented no lesions, 10-25% represented mild lesions, 25-50% represented moderate lesions, 50-75% represented severe lesions, and 75-100% represented critical lesions. Using a microscope eyepiece camera (MD500, 2019) and image analyzer software (AmScope, 3.7), lesion scoring was computed semi-quantitatively. A light microscope was used for the examination of tissue samples.

### Statistical Analysis

GraphPad Prism 8 (GraphPad Software, San Diego, CA, USA) was used for the statistical analysis. The data was presented as SD ± mean. One-way analysis of variance (ANOVA) was utilized for group comparisons, and the Tukey test was applied for multiple comparisons. To compare each group with the ethanol-treated group, unpaired *t*-tests were employed. Statistical significance was attained when the *P*-value was (<.05) in the obtained results.

## Results

**Impact of Quercetin, Misoprostol, and Their Combination on Stomach Lesion Area, Lesion Index, and Lesion Inhibition**

The gastric lesion area was decreased significantly (*P*-value < .05) by each of esomeprazole, quercetin, misoprostol, and combination therapy in comparison with the ethanol-treated group. The ulcer index also showed significant attenuation in the esomeprazole, quercetin, and the combination groups, with the maximum amelioration achieved by the combination group, which was similar to that produced by esomeprazole. Regarding the percentage of ulcer inhibition; the use of quercetin resulted in (38.84%) inhibition, while misoprostol produced only (26%) inhibition. The use of misoprostol with quercetin resulted in maximum protection (40.98%), resembling that achieved by esomeprazole (41.64%) (Table 1).

**Impact of Quercetin, Misoprostol, and Their Combination on the Parameters of Gastric Juice**

**Figure 1.**
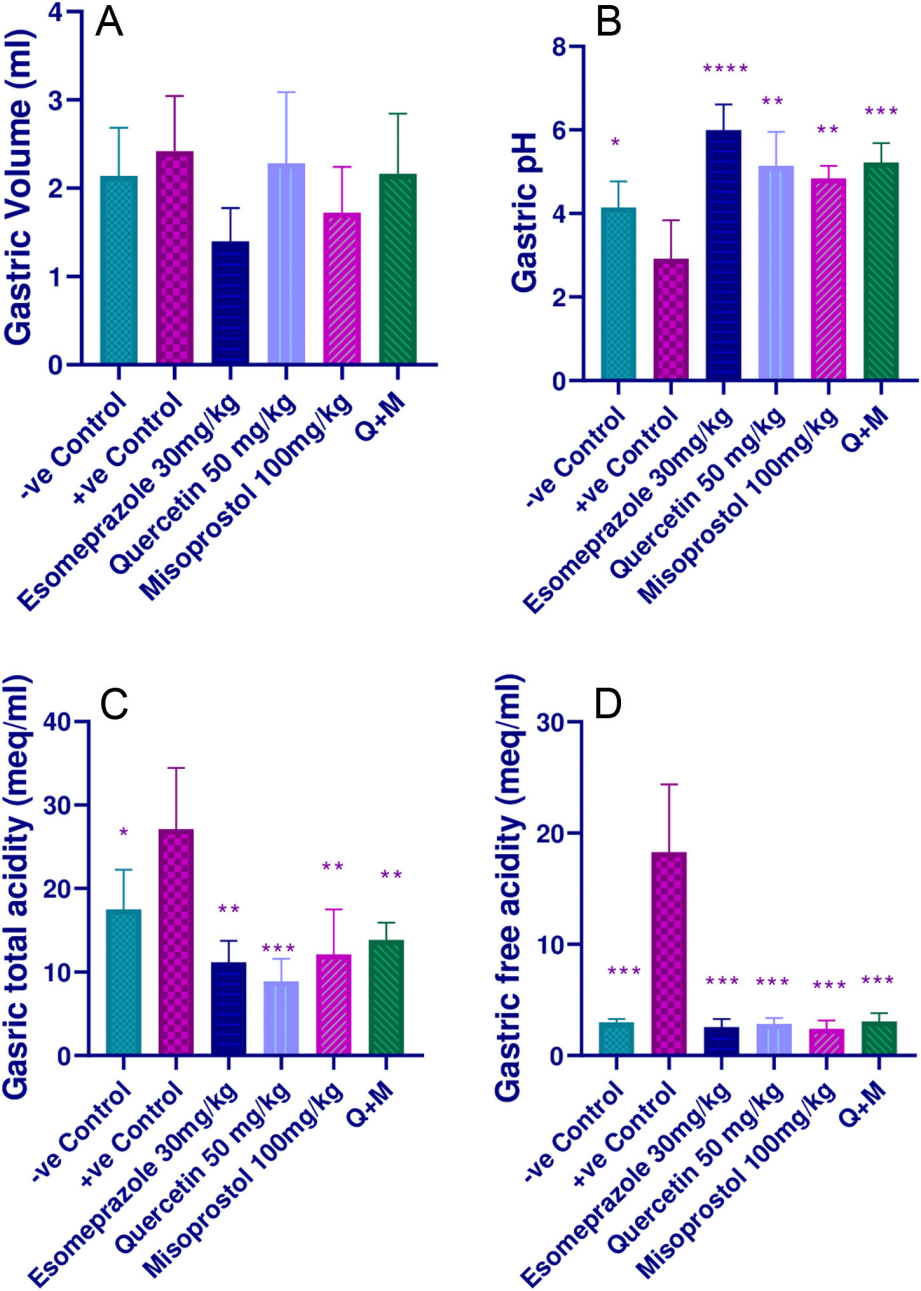
Effect of quercetin and misoprostol on (A) the gastric volume, (B) gastric pH, (C) gastric total acidity, and (D) gastric free acidity; * (*P* < .05), ** (*P* < .01), ***(*P* < .001), and **** (*P* < .0001) significantly different compared to the positive control group using one-way ANOVA and an unpaired *t*-test.

The study showed no significant change between the groups (*P*-value > .05) (Figure 1A). The negative control revealed a significant increase in the pH of the gastric juice (*P*-value < .05). The esomeprazole-treated group and the combination group showed comparable effects that were significantly higher than the ethanol-treated group (*P*-value < .0001) and (*P*-value < .001), respectively. Both quercetin and misoprostol alone also produced a significant elevation in gastric pH values in comparison with the positive control group (*P*-value < .01); however, their effects alone were less than those produced by their combination when compared to the esomeprazole-treated group (*P*-value < .05) (Figure 1B).

The attenuation observed in the levels of total and free acidity of the negative control group was statistically significant (*P*-value < .05) and (*P*-value < .001, respectively) when compared to the ethanol-treated group. The esomeprazole-treated group also reduced the levels of total and free acidity significantly (*P*-value < .01 and *P*-value < .001). All the treatment groups produced significant reductions in free acidity (*P*-value < .001) and total acidity (*P*-value < .01) as compared to the ethanol-treated group. Additionally, the combination group and misoprostol group produced a parallel effect to the esomeprazole group regarding free and total acidity (*P*-value > .05), while the quercetin alone group decreased the total acidity more than the esomeprazole group (*P*-value < .05) (Figures 1C and D).

**Impact of Quercetin, Misoprostol, and Their Combination on LDH and IL-6**

**Figure 2.**
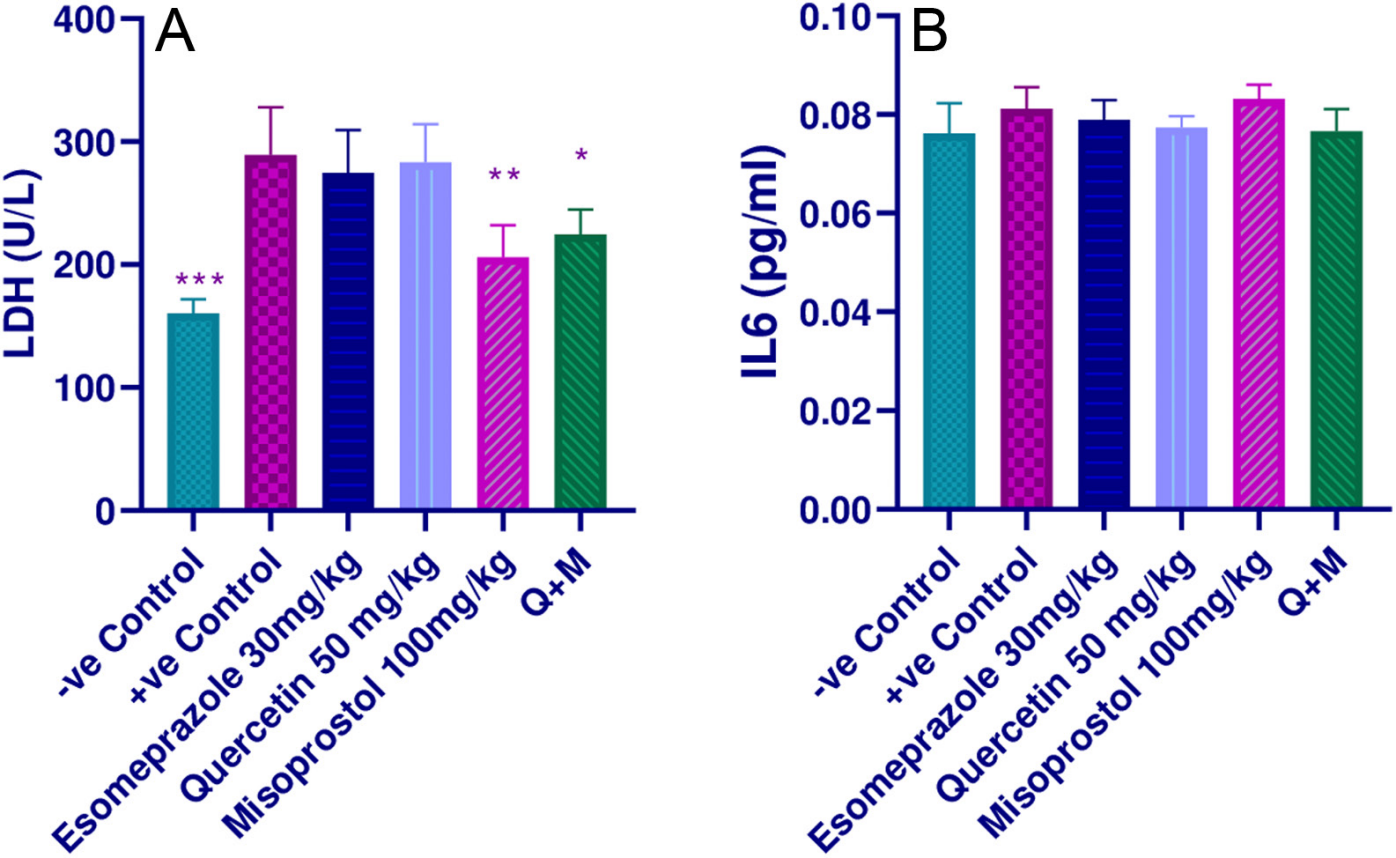
Effect of quercetin and misoprostol on (A) LDH, and (B) IL-6; * (*P* < .05), ** (*P* < .01), and *** (*P* < .001) significantly different compared to the positive control group using one-way ANOVA and an unpaired *t*-test.

The LDH levels elevated significantly in the ethanol-treated group (*P* value< .001). While its level was decreased in the treated groups, only misoprostol (*P-*value < .01) and the combination group, (*P*-value< .05) reached a significant level when compared to both the positive control and esomeprazole groups (Figure 2A). Regarding the serum IL-6 level, the combination and misoprostol groups non-significantly attenuated its level (*P*-value > .05) (Figure 2B).

**Impact of Quercetin, Misoprostol, and Their Combination on Serum Catalase and GSH**

**Figure 3.**
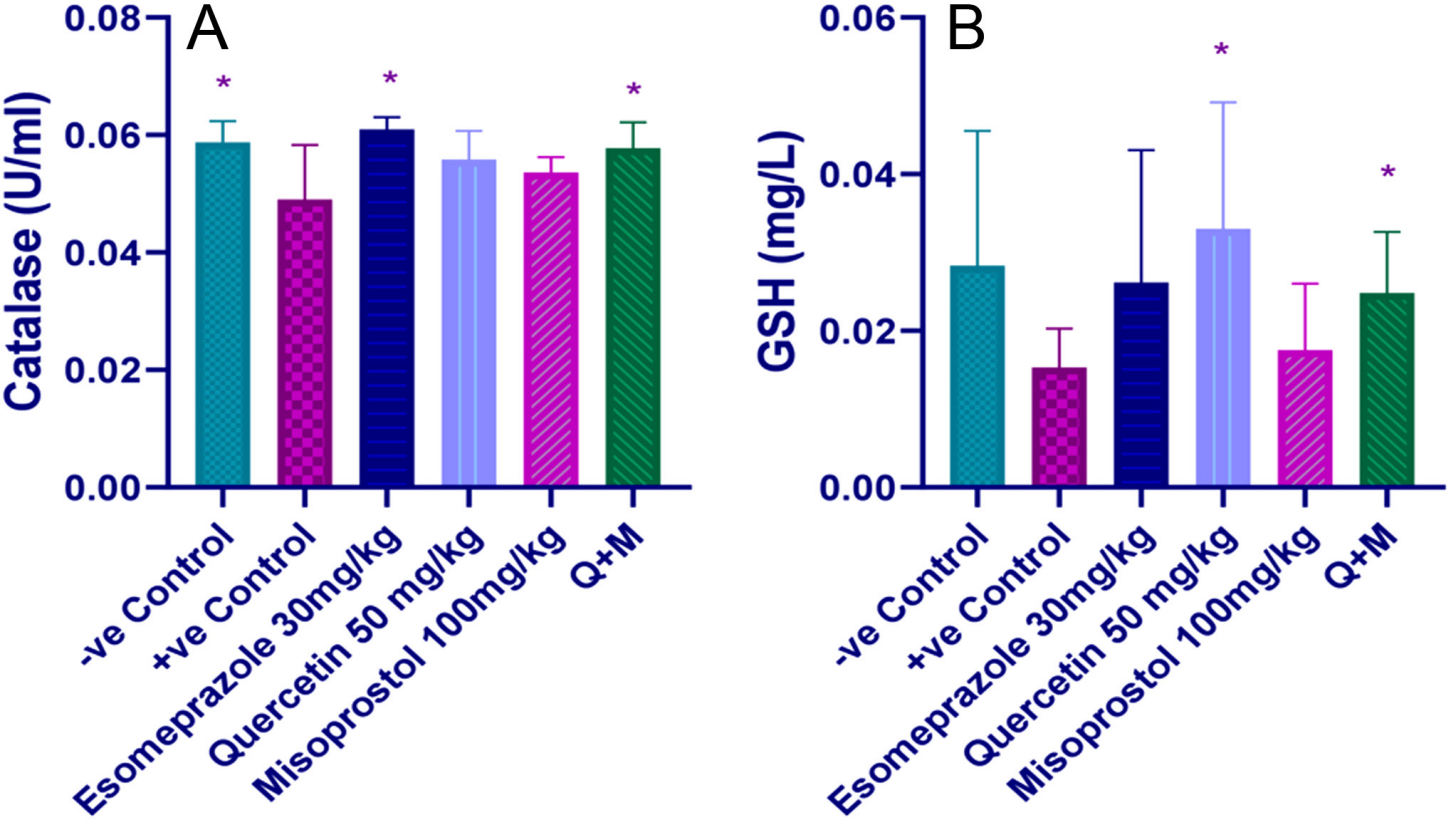
Effect of quercetin and misoprostol on (A) Catalase and (B) GSH; * (*P* < .05) significantly different compared to the positive control group using one-way ANOVA and an unpaired *t*-test.

Catalase levels decreased significantly in ethanol treated group, (*P*-value< .05). Esomeprazole and the combination therapy successfully and significantly elevated the levels (*P*-value < .05). Quercetin and misoprostol groups, each alone increased the level of catalase; however, they did not reach a significant level when compared with both the positive control and esomeprazole groups (*P*-value > .05), (Figure 3A). Serum GSH attenuated in the positive control group and was restored by both quercetin and the combination groups when compared to both the positive control and esomeprazole groups (*P*-value < .05) (Figure 3B).

### Gross Evaluation of Gastric Lesions

**Figure 4.**
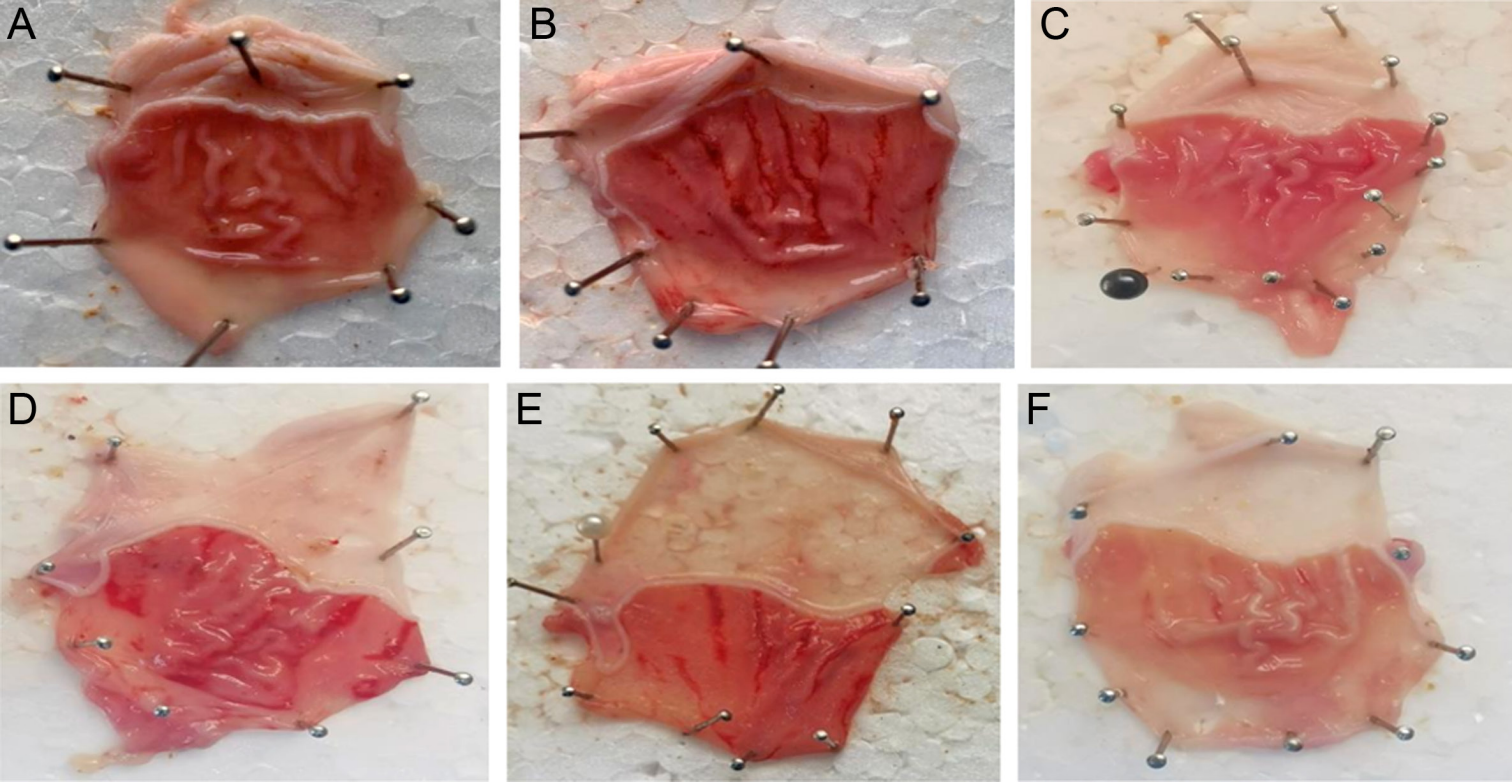
Representative images of stomachs from experimental groups: (A) negative control group, (B) ethanol-treated group, (C) esomeprazole-treated group (30 mg/kg), (D) quercetin-treated group (50 mg/kg), (E) misoprostol-treated group (100 mg/kg), and (F) quercetin + misoprostol-treated group.

Macroscopic analysis shows that misoprostol and quercetin have a gastroprotective effect, with the combination group achieving the highest level of protection. The pictures of the 6 treatment groups are shown in Figure 4A–F. In comparison with the ethanol-treated group (Figure 4B), the antiulcer efficacy of esomeprazole (Figure 4C) and combination therapy (Figure 4F) was similar and easily visible.

### Histopathological Findings

**Figure 5.**
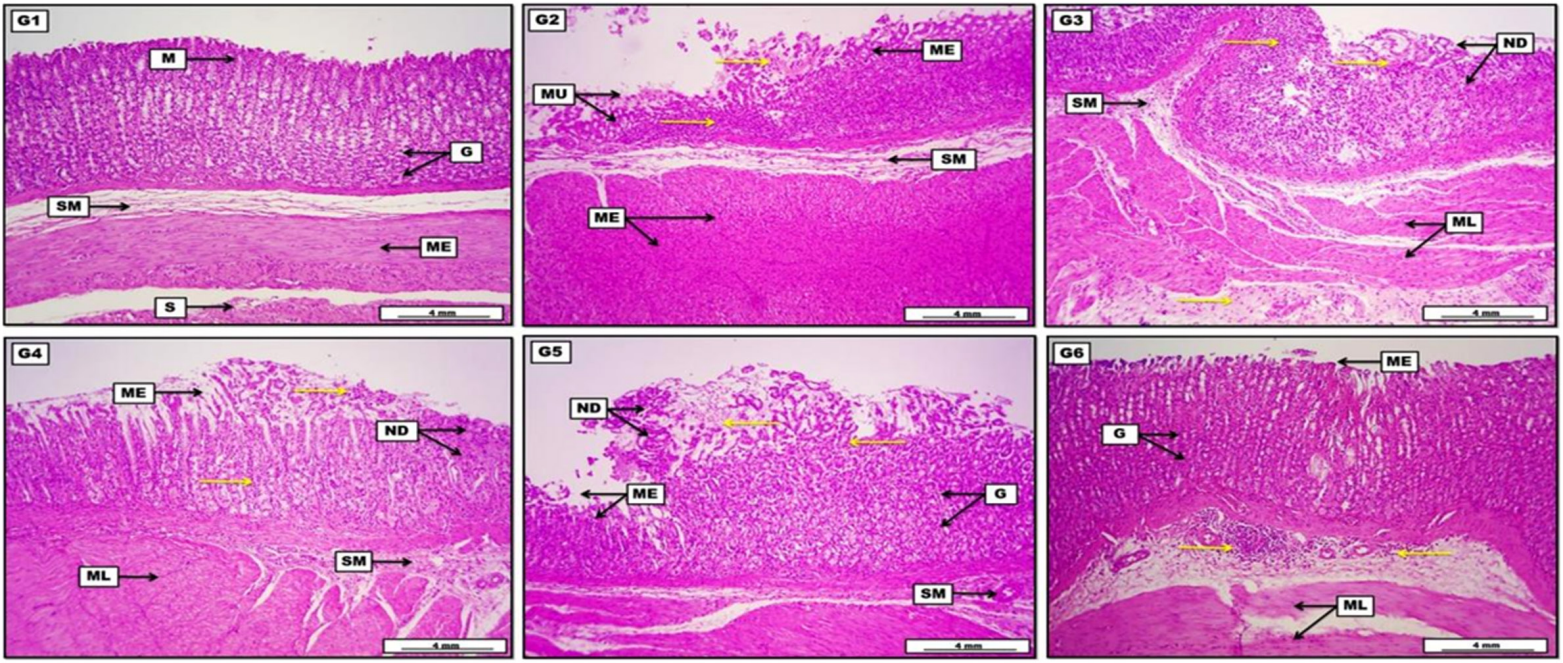
Photomicrograph of the stomach from groups: (G1): The negative control group showed no apparent morphological changes, evident in undamaged and typically appeared gastric layers starting with the first mucosal layer (M) with normally arranged gastric glands (G), the normal loose connective tissue of submucosa (SM), double layers of muscular external smooth muscle (ME), and the outermost layer of serosa (S). (G2): The positive ulcer-control group demonstrated the presence of clear mucosal ulceration (MU) and a wide area of mucosal erosion (ME), together with the presence of diffusely distributed pinkish inflammatory exudates (yellow arrows) mixed with deep eosinophilic necrotic debris. The inflammatory cells also infiltrated the submucosal layer (SM), with significant hyperplasia of the muscularis externa (ME). (G3): The Esomeprazole treatment group revealed the presence of acidophilic necrotic debris (ND) at the erosion surface mixed with a few inflammatory exudates and inflammatory cells (yellow arrows). The inflammatory exudates also infiltrated diffusely within the submucosal connective tissue (SM) and even in between the muscle layers (ML). (G4): The quercetin group showed the presence of light pinkish inflammatory exudate (yellow arrows) together with deep eosinophilic necrotic debris (ND). The gastric mucosa reveals areas of mucosal erosion (ME) and other areas of glandular regeneration (G). Moreover, the submucosa (SM) contains a low grade of inflammatory exudate. (G5): The misoprostol treatment group displayed the presence of superfine light-pinkish inflammatory exudates (yellow arrows) mixed with mucosal necrotic debris (ND) in the area of mucosal erosion (ME). The inflammatory exudates were also diffused among the gastric glands and within the submucosal layer (SM), together with the obvious thickening of the muscular layers (ML). (G6): The misoprostol and quercetin group illustrated a significant reduction in the area of mucosal erosion (ME), together with considerable mucosal glandular regeneration (G), in addition to the low grade of submucosal inflammatory cell infiltration (yellow arrows). The outer muscular layers (ML) appeared typically arranged and unharmed. H&E. Scale bar: 4 mm.

First and foremost, Table 2 illustrates in detail the semi-quantitative morphometric evaluation of gastric sections’ lesion-scoring mechanism. In a general scene, oral force-feeding of 1 mL of 90% ethanol will induce serious damage and critical erosion to the gastric mucosa within the first few hours of administration, evident by acute mucosal sloughing, accumulation of edematous fluid mixed with inflammatory exudates and necrotic debris together with substantial infiltration of inflammatory cells, as shown in Figure 5, in respect to the positive control group G2. Furthermore, treatment groups exhibited significant *P*-value <.05 alleviation in the lesion severity according to the experimental groups. Surprisingly, animals treated with a conjunction preventive measure of misoprostol and quercetin, in a dose of 100 and 50 mg/kg, respectively, proved very significant mitigation in the lesion severity from critical to moderate in comparison to the positive ulcer group G2, explained by significant mitigation in the inflammatory exudates along with distinct regeneration of the severed tissues and subsiding of the inflammatory reaction. On the other hand, a daily prophylactic remedy for 14 days with the ordinary antacid esomeprazole in a dose of 30 mg/kg (G3) showed a reduction in lesion scoring. The quercetin-treated group of 50 mg/kg (G4) showed a significant decrease in the lesion compared to the ethanol-treated group; however, lesion grading ranged between moderate to severe. Additionally, the misoprostol-treated group (G5) in a dose of 100 mg/kg disclosed a significant attenuation in the lesion severity as compared to the ethanol-treated and quercetin groups. In conclusion, tissue sections from G3, G5, and G6 treatment groups show significant improvement in the scoring of the lesion when compared to other treatment groups, although the results are much more significant in the misoprostol and quercetin group (G6).

## Discussion

Nutraceuticals are a growing option for the management of gastrointestinal (GI) diseases since they are considered to be safe when compared to pharmaceutical agents used for the same purposes.^14^ Quercetin is one of the flavonoids that gained pronounced attention in the last decade due to its well-known antioxidant and anti-inflammatory properties.^15^ Studies showed the beneficial effects of quercetin on improving GI functions^16,17^ and attenuating myeloperoxidase levels, which is used as an indicator for neutrophil infiltration and has the ability to generate reactive species triggering oxidative stress.^18^ Therefore, the use of quercetin could be of value in protecting gastric mucosa from the deleterious effects caused by oxidative damage produced by ethanol. Various studies proved the importance of combining drugs with quercetin in different animal models and the outcomes were promising.^19-22^ Accordingly, this study aimed to assess the possible synergistic effect of adding quercetin to misoprostol. This combination resulted in significant protection against gastric ulcers induced by ethanol, which was clearly manifested by the percentage of ulcer inhibition in a parallel manner with the inhibitory effects offered by esomeprazole. Moreover, a remarkable elevation in gastric pH and a significant reduction in both free and total acidity were also observed in the combination group. The antiapoptotic effect produced by quercetin may be attributed to such an effect.^17^ Additionally, the gastroprotective effect produced by misoprostol through decreasing acid production in the gut and elevating prostaglandin levels, which protects the gastric mucosa from any deleterious effects of gastric irritants, also contributes to such protection.^23^ Furthermore, the antioxidant markers used in the present study were significantly reduced by ethanol, as one of the mechanisms of ethanol-induced gastric ulcer is mediated via generating reactive species, and adding quercetin was effective in restoring the levels of antioxidant enzymes. Part of the protective effect of quercetin is related to inhibiting lipid peroxidation^24^ and boosting total antioxidant capacity^19^ by regulating the level of glutathione^25^ and antioxidant enzymes.^26^ In the present study, LDH and IL-6 increased in the ethanol-treated group. LDH levels usually increase during gastric ulcers and can be used as an indicator of the presence of injury.^27^ IL-6 levels were also shown to increase during gastric ulcers.^28^ The anti-inflammatory effect of quercetin can contribute to the protective effect of quercetin, as studies revealed the involvement of quercetin in suppressing proinflammatory cytokines.^29^ On the other hand, misoprostol proved to mitigate inflammation,^30^ and combining quercetin with misoprostol resulted in a better attenuation of LDH and IL-6 levels compared to the use of each alone. Finally, macroscopic and microscopic findings greatly support the biochemical findings, with a clear alleviation in the area of lesion, edema, and inflammatory exudate observed in the combination group, which was comparable to that observed with esomeprazole. Additionally, this combination was also successful in attenuating necrotic debris, mucosal erosion, and inflammatory cells. The limitation of the current study could be attributed to the inability to measure the antioxidant and inflammatory parameters in the gastric tissue, since fixing and picturing the gastric tissues for measuring the ulcer index exposed the tissues to light for a long period, and this may result in unreliable data if the aforementioned parameters are measured in the tissue.

## Conclusion

This study clearly revealed the gastroprotective effect produced by combining quercetin with misoprostol by decreasing ulcer area and index, restoring antioxidant enzyme levels, and ameliorating inflammation. These findings propose this combination to be used in a clinical setting.

## Figures and Tables

**Table 1.** Impact of Quercetin, Misoprostol, and Their Combination on Gastric Ulcer Area, Gastric Ulcer Index, and Percentage of Lesion Inhibition

Experimental Groups, n = 5	Total Gastric Area (mm^2^) (Mean)*	Ulcer Area (mm^2^) (Mean)*	Ulcer Index (Mean)*	% of Inhibition
Positive control	69	4.4^A^#	6.3^A^	
Esomeprazole 30 mg/kg	43.8	0.43^B^	1^B^	41.64
Misoprostol 100 mg/kg	49.69	2.57^C^	5.1^A^	26.7
Quercetin 50 mg/kg	53.4	1.99^D^	3.7^C^	38.84
Quercetin + misoprostol	64.5	1.3^D^	2^B^	40.98

Notes: *Each value in the gastric lesion study was approximated as the mean average of 5 animals (n = 5). #Comparing groups statistically: Significant differences are found between mean values with different capital letters at (*P*-value < .05).

**Table 2.** Morphometric Semiquantitative Evaluation of Gastric Tissue Sections

Experimental Groups, n = 5	Mucosal Erosion (Mean%)*	Edema and Inflammatory Exudate (Mean%)*	Inflammatory Cells (Mean%)*	Necrotic Debris (Mean%)*	Lesion Scoring (0-100%)	Lesion Grading
Negative control	1.56%^A^#	4.37%^A^	5.89%^A^	3.12%^A^	0%-10%	No lesion
Positive control	95.64%^E^	92.28 %^E^	94.72%^E^	84.49% ^E^	75%-100%	Critical
Esomeprazole 30 mg/kg	43.11%^C^	44.86%^C^	46.52%^C^	44.73%^C^	25%-50%	Moderate
Quercetin 50 mg/kg	62.41%^D^	67.93%^D^	72.68%^D^	66.38%^D^	50%-75%	Severe
Misoprostol 100 mg/kg	48.32%^C^	44.39%^C^	42.78%^C^	41.58%^C^	25%-50%	Moderate
Quercetin + misoprostol	27.89%^C^	31.56%^C^	39.71%^C^	26.37%^C^	25%-50%	Moderate

Notes: *Each value in the morphometric lesion scoring analysis indicates the mean percentage (%) of 5 animals (n = 5). All morphometric values were approximated as mean averages. #Comparing groups statistically: Significant differences are found between mean values with various capital letters at (*P*-value < .05).

## Data Availability

The data that support the findings of this study are available on request from the corresponding author.

## References

[1] Mota KSL Dias GEN Pinto MEF , et al. Flavonoids with gastroprotective activity. Molecules. 2009;14(3):979 1012. (10.3390/molecules14030979) 19305355 PMC6253827

[2] Kumar V Abbas A Aster J . Robbins Basic Pathology E. 10th ed. Elsevier Health Sciences; Amsterdam; 2017.

[3] Scida S Russo M Miraglia C , et al. Relationship between Helicobacter pylori infection and GERD. Acta Biomed. 2018;89(8-S):40 43. (10.23750/abm.v89i8-S.7918) PMC6502218 30561416

[4] Bafna PA Balaraman R . Anti-ulcer and antioxidant activity of DHC-1, a herbal formulation. J Ethnopharmacol. 2004;90(1):123 127. (10.1016/j.jep.2003.09.036) 14698519

[5] Vonkeman HE Klok RM Postma MJ Brouwers JRBJ Van De Laar MAFJ . Direct medical costs of serious gastrointestinal ulcers among users of NSAIDs. Drugs Aging. 2007;24(8):681 690. (10.2165/00002512-200724080-00005) 17702536

[6] Massignani JJ Lemos M Maistro EL , et al. Antiulcerogenic activity of the essential oil of Baccharis dracunculifolia on different experimental models in rats. Phytother Res. 2009;23(10):1355 1360. (10.1002/ptr.2624) 19274697

[7] Repetto MG Llesuy SF . Antioxidant properties of natural compounds used in popular medicine for gastric ulcers. Braz J Med Biol Res = Rev Bras Pesqui medicas e Biol. 2002;35(5):523 534. (10.1590/s0100-879x2002000500003) 12011936

[8] Coşkun Ö Kanter M Armutçu F Çetin K Kaybolmaz B Yazgan Ö . Protective effects of quercetin, a flavonoid antioxidant. In: Absolute Ethanol-Induced ACUT Gastric Ulcer. Electron J Gen Med.; 1(3); 2004:37 42.

[9] Wood AJJ Walt RP . Misoprostol for the treatment of peptic ulcer and antiinflammatory-drug–induced gastroduodenal ulceration. N Engl J Med. 1992;327(22):1575 1580. (10.1056/NEJM199211263272207) 1435885

[10] Kim YS Nam YS Song J Kim H . Gastroprotective and healing effects of polygonum cuspidatum root on experimentally induced gastric ulcers in rats. Nutrients. 2020;12(8):1 15. (10.3390/nu12082241) PMC7468921 32727104

[11] Hama Amin RR Aziz TA . Gastroprotective effect of azilsartan through ameliorating oxidative stress, inflammation, and restoring hydroxyproline, and gastrin levels in ethanol-induced gastric ulcer. J Inflamm Res. 2022;15:2911 2923. (10.2147/JIR.S365090) 35592072 PMC9113664

[12] Rao G Sujatha D Rupa V Priya E Rao K Venkateswarlu M . Study on anti-ulcer activity of Daucus carota juice. Res J Pharm Tech. 2010;3(2):547 550.

[13] Ketuly KA Abdulla MA Hadi HA Mariod AA Ibrahim S . Wahab A-. Anti-ulcer activity of the 9alpha-bromo analogue of beclometha­sone dipropionate against ethanol-induced gastric mucosal injury in rats. J Med Plants Res. 2011;5(4):514 520.

[14] Gao X Liu J Li L Liu W Sun M . A brief review of nutraceutical ingredients in gastrointestinal disorders: evidence and suggestions. Int J Mol Sci. 2020;21(5). (10.3390/ijms21051822) PMC7084955 32155799

[15] Carullo G . Quercetin and its natural sources in wound healing management. Curr Med Chem. 2018;25(June):1 22. 10.2174/0929867325666180713150626 30009700

[16] Feng J Li Z Ma H , et al. Quercetin alleviates intestinal inflammation and improves intestinal functions via modulating gut microbiota composition in LPS-challenged laying hens. Poult Sci. 2023;102(3):102433. (10.1016/j.psj.2022.102433) 36587451 PMC9816806

[17] Abdel-Tawab MS Mostafa Tork O Mostafa-Hedeab G Ewaiss Hassan M Azmy Elberry D . Protective effects of quercetin and melatonin on indomethacin induced gastric ulcers in rats. Rep Biochem Mol Biol. 2020;9(3):278 290. (10.29252/rbmb.9.3.278) 33649721 PMC7816780

[18] Kahraman A Erkasap N Köken T Serteser M Aktepe F Erkasap S . The antioxidative and antihistaminic properties of quercetin in ethanol-induced gastric lesions. Toxicology. 2003;183(1-3):133 142. (10.1016/s0300-483x(02)00514-0) 12504347

[19] Aziz TA . Cardioprotective effect of quercetin and sitagliptin in doxorubicin-induced cardiac toxicity in rats. Cancer Manag Res. 2021;13:2349 2357. (10.2147/CMAR.S300495) 33737832 PMC7965691

[20] Sharma S Cwiklinski K Mahajan SD Schwartz SA Aalinkeel R . Combination modality using quercetin to enhance the efficacy of docetaxel in prostate cancer cells. Cancers (Basel). 2023;15(3). (10.3390/cancers15030902) PMC9913446 36765857

[21] Ali HH Ahmed ZA Aziz TA . Effect of telmisartan and quercetin in 5 fluorouracil-induced renal toxicity in rats. J Inflamm Res. 2022;15:6113 6124. (10.2147/JIR.S389017) 36386583 PMC9651059

[22] Ahmed ZA Abtar AN Othman HH Aziz TA . Effects of quercetin, sitagliptin alone or in combination in testicular toxicity induced by doxorubicin in rats. Drug Des Devel Ther. 2019;13:3321 3329. (10.2147/DDDT.S222127) PMC6759798 31571833

[23] Krugh M Maani CV . Misoprostol. xPharm Compr Pharmacol Ref. Published online April 21, 2023:1 5.

[24] Scholten SD Sergeev IN Scholten S . Long-term quercetin supplementation reduces lipid peroxidation but does not improve performance in endurance runners. Open Access J Sports Med. 2013;4:53 61. (10.2147/OAJSM.S39632) 24379709 PMC3871649

[25] Xu D Hu MJ Wang YQ Cui YL . Antioxidant activities of quercetin and its complexes for medicinal application. Molecules. 2019;24(6):1123. (10.3390/molecules24061123) 30901869 PMC6470739

[25] Qi W Qi W Xiong D Long M . Quercetin: Its antioxidant mechanism, antibacterial properties and potential application in prevention and control of toxipathy. Mol. 2022;27(19):6545. 10.3390/molecules27196545 PMC9571766 36235082

[27] Teniola D Ayoola EA Arigbabu AO . Lactic dehydrogenase levels in patients with duodenal ulcer, gastric ulcer, gastric polys and gastric carcinoma. Scand J Gastroenterol Suppl. 1986;124(S124):169 178. (10.3109/00365528609093801) 3508633

[28] Omar SA Hassan MF Hasan BB . Impact of Serum interleukin 6 among Helicobacter Pylori-Positive Adult Patients in Relation to upper gastrointestinal endoscopy findings. Med J. Suez Canal Univ. 2019;22(2):117 121. (10.21608/scumj.2019.94803)

[29] Rizky WC Jihwaprani MC Mushtaq M . Protective mechanism of quercetin and its derivatives in viral-induced respiratory illnesses. Egypt J Bronchol. 2022;16(1):1 8. (10.1186/s43168-022-00162-6)

[30] Doube A Davies J Notarianni L Holgate K Fenn GC . Effect of misoprostol on concentrations of prostaglandins in synovial fluid. Ann Rheum Dis. 1991;50(11):797 799. (10.1136/ard.50.11.797) 1772296 PMC1004561

